# Combining External Beam Radiotherapy and Immunotherapy for the Treatment of Hepatocellular Carcinoma

**DOI:** 10.3390/curroncol33040226

**Published:** 2026-04-17

**Authors:** Connie Le, Aswin G. Abraham, Keith Tankel, Nawaid Usmani, Kurian Joseph, Diane Severin, Fatimah AlFaraj, Laura A. Dawson

**Affiliations:** 1 Department of Oncology, Division of Radiation Oncology, University of Alberta, Edmonton, AB T6G 1Z2, Canadakurian.joseph@albertahealthservices.ca (K.J.);; 2 Cross Cancer Institute, Edmonton, AB T6G 1Z2, Canada; 3 Radiation Medicine Program, Princess Margaret Cancer Centre, Toronto, ON M5G 2M9, Canada; laura.dawson@uhn.ca; 4 Department of Radiation Oncology, University of Toronto, Toronto, ON M5T 1P5, Canada

**Keywords:** hepatocellular carcinoma, radiotherapy, immunotherapy, macrovascular invasion, PD-1, PD-L1, CTLA-4, angiogenesis inhibitor, tyrosine kinase inhibitor, stereotactic body radiotherapy

## Abstract

The advent of immune checkpoint inhibitors has driven progress in hepatocellular carcinoma (HCC) treatment outcomes and enabled opportunities for combining therapeutic modalities. Growing evidence substantiates the utility of radiotherapy, particularly at ablative doses, in the management of HCC. Given the potential for radiotherapy to induce an immunostimulatory environment and potentiate immune checkpoint inhibitor activity, the expanding HCC treatment landscape compels exploration of the combination of radiotherapy and immunotherapy. This review highlights recent advances in the treatment of HCC using radiotherapy and immunotherapy in combination. Radiation can potentiate an anti-tumor response and tumor microenvironment permissive to immunotherapy. Results from randomized clinical trials and retrospective studies consistently show that combinations of radiotherapy and immunotherapy improved the treatment outcomes of unresectable or advanced HCC—especially HCC with macrovascular invasion. Active research to further improve treatment efficacy and reduce side effects is exemplified by more than 20 ongoing clinical trials combining external beam radiotherapy and immunotherapy to treat HCC. Ongoing research aims at prolonging survival and downstaging advanced or unresectable HCC.

## Simple Summary

Hepatocellular carcinoma (HCC) is the sixth most common solid organ cancer and the third leading cause of cancer-related death worldwide. HCC is a radiosensitive tumor, and radiation can stimulate an anti-tumor response and create a tumor microenvironment favorable to immunotherapy. This review highlights recent advances in the treatment of HCC using radiotherapy and immunotherapy in combination. Randomized clinical trials and retrospective studies have shown that combinations of radiotherapy and immunotherapy improved the treatment outcomes of unresectable or advanced HCC. More than 20 ongoing clinical trials combining external beam radiotherapy and immunotherapy to treat HCC aim at further improving safety, prolonging survival, and downstaging advanced or unresectable HCC.

## 1. Introduction

Hepatocellular carcinoma (HCC) is a notable global health burden as the sixth most common solid organ cancer and the third leading cause of cancer-related death worldwide [1]. The global mortality to incidence ratio of HCC is 0.98, suggesting that the great majority of people with HCC die from their malignancy [2]. The high HCC lethality is compounded by comorbidities, such as cirrhosis and chronic hepatitis viral infection. Furthermore, HCC is predominantly diagnosed at advanced or metastatic stages because of the general lack of symptoms in early stages of the cancer [3]. In the United States, the 5-year overall survival (OS) rate of patients with HCC is approximately 20% and deteriorates to 3% with distant metastases at presentation [4]. The global 5-year OS of patients with HCC is approximately 10%. Notably, both the incidence and mortality rate of HCC have been rising in North America [5,6,7], underscoring the unmet need for innovations in HCC therapy regimens.

Treatment approaches are directed by the stage of diagnosis and the patient’s eligibility for surgical intervention. The Barcelona Clinic Liver Cancer (BCLC) staging system is widely used for directing therapy and prognostication for HCC cases [8,9]. The BCLC staging system accounts for tumor burden, liver function, performance status, and tumor macrovascular invasion (MVI). HCC stages are classified as 0 (very early stage), A (early stage), B (intermediate stage), C (advanced stage), or D (terminal stage). In early-stage patients, surgery (resection or liver transplantation) is the preferable intervention. In patients not amenable to or not suitable for surgery with liver-confined HCC, locoregional therapies, such as external beam radiotherapy (EBRT), radiofrequency ablation (RFA), transarterial radio-embolization (TARE), and transarterial chemoembolization (TACE), can be used in selected patients. Advanced-stage HCC is a heterogenous group that includes patients with MVI or extrahepatic disease. Systemic therapies are standard for advanced stage HCC or HCC unsuitable for surgical, ablative, or transarterial modalities.

Overall, HCC management has been a dynamic field with evolving applications for radiotherapy and systemic therapies. In 2008, sorafenib, a tyrosine kinase inhibitor (TKI), became the first globally approved systemic therapy for unresectable or metastatic HCC [10]. Almost a decade later with the introduction of immune checkpoint inhibitors (ICIs), numerous immunotherapy regimens improved survival when compared with TKIs, making immunotherapy the new standard of care systemic therapy (Table 1 and Table 2). In this review, we highlight recent advances in radiotherapy, immunotherapy, and their combination in the treatment of HCC.

## 2. Radiotherapy for Hepatocellular Carcinoma

HCC is a radiosensitive tumor. With advances in the accuracy and precision of radiotherapy, stereotactic body radiation therapy (SBRT) involving highly conformal EBRT at ablative doses can be delivered safely [11]. SBRT confers improved outcomes, including objective response rate (ORR), overall survival (OS), and progression-free survival (PFS), compared to conventionally fractionated EBRT [12]. The 2-year local control rates following SBRT ranged from 75% to over 90%, depending on the size of the lesion and the radiation dose delivered [13,14]. Recent expert consensus has helped define ablative EBRT in the treatment of HCC [15].

Randomized control trials have substantiated EBRT as a locoregional treatment for non-operative HCC. In a randomized control trial of SBRT versus RFA in patients with a single recurrent HCC lesion ≤ 5 cm, SBRT improved the 2-year local PFS to 92.7% from 75.8% (*p* = 0.014) [16]. OS was also improved, although these findings were not statistically significant [16]. A randomized phase III trial compared proton beam radiotherapy with RFA in 144 patients with recurrent or residual HCC [17]. The 2-year local PFS was 94.8% in patients receiving proton beam radiotherapy compared to 83.9% (*p* = 0.001) for patients treated with RFA [17]. Proton beam radiotherapy also showed improved local control (hazard ratio (HR) 5.64, *p* = 0.003) and PFS (HR 3.62, *p* = 0.002) compared to TACE in a phase III randomized trial of transplant eligible patients with no prior treatment [18]. Randomized clinical trials have shown improved 1-year local control (84% vs. 23%, *p* = 0.0002) [19] and time to local recurrence (>40 months vs. 12 months) [20] when comparing SBRT and TACE. Another phase III randomized control trial demonstrated improved median local control (from 13.1 months to not reached, *p* < 0.001), improved but not statistically significant median PFS (from 11.6 to 15.4 months, *p* = 0.07), and median OS (from 36.8 to 47.1 months, *p* = 0.65) with the addition of EBRT to TACE compared to TACE alone [21]. The favorable local control of SBRT may be related to tumor vasculature and resource variability influencing TACE efficacy. The randomized control trials comparing EBRT and TACE did not show significantly different OS, being limited by small sample sizes from early trial closures and poor accrual. Salvage SBRT was also effective following incomplete TACE in a single-arm phase II trial with a 2-year local control rate of 94.6% and 2-year OS of 68.7% [22].

Meta-analyses and retrospective studies also support the utility of EBRT for the treatment of HCC. Meta-analyses have shown similar or improved HCC local control rates in patients undergoing SBRT compared to RFA [23,24,25,26]. A meta-analysis of retrospective studies demonstrated statistically improved local control yet inferior OS of SBRT versus RFA [27]. Retrospective analyses have also established at least comparable local recurrence risk with SBRT compared to RFA [28,29,30]. Various factors including tumor size, number, and location in different liver segments impact the efficacy of SBRT and RFA. For instance, a retrospective study found SBRT conferred better local control for segment 7 and 8 tumors, whereas RFA led to improved OS for segment 5 and 6 lesions [31]. A meta-analysis comparing EBRT and TACE for upfront HCC treatment demonstrated improved local control (HR 0.16) and PFS (HR 0.37) with EBRT, and there was no significant difference in OS and toxicity profile [32]. Retrospective studies showed similar or better local control and OS when HCC lesions were treated with SBRT compared to TACE [33,34]. Several investigations and meta-analyses have consistently shown improved OS with combined EBRT and TACE compared to TACE monotherapy in unresectable HCC [35,36,37]. SBRT followed by TACE has also conferred improved OS and similar PFS compared to SBRT alone [38]. Radiation is also an effective alternative to other locoregional therapies in the salvage treatment of recurrent or residual HCC [39,40].

SBRT is also efficacious in treating MVI, which is found at the time of diagnosis in approximately 35% to 50% of patients with HCC [41]. MVI is an independent poor prognostic factor that is associated with median OS of 2–5 months with best supportive care [42]. MVI can increase the risk of disseminating metastases and cause additional pathology, such as portal hypertension, ascites, and liver failure. By disrupting hepatic circulation, MVI can also worsen prognosis by reducing the efficacy of therapies reliant on patent vasculature. In a randomized control trial in patients with HCC MVI, TACE with EBRT conferred favorable outcomes compared with sorafenib. The TACE-radiotherapy arm experienced significantly improved 12-week PFS (86.7% vs. 34.3%, *p* < 0.001), radiologic response rate (33.3% vs. 2.2%, *p* < 0.001), downstaging enabling resection (11.1% vs. 0%), and median OS (55 vs. 43 weeks, *p* = 0.04) [43]. Another randomized trial assessed patients with resectable HCC with MVI who were treated with pre-operative EBRT or upfront surgery. The neoadjuvant radiotherapy arm had significantly improved OS (27.4% vs. 9.4% at 24 months, *p* < 0.001), disease-free survival (13.3% vs. 3.3% at 24 months, *p* < 0.001), HCC-related mortality (HR 0.35, *p* < 0.001), and recurrence rates (HR 0.45, *p* < 0.001) compared with surgery alone [44]. Retrospective analyses have reported 1-year local control ranging from 80% to 87.4%, a median OS of 18.3 months, and 1-year OS ranging from 30% to 60% [45,46,47,48]. In a propensity score-matched analysis of HCC patients with MVI who underwent surgery or SBRT, both groups had an OS of 16 months (*p* = 0.54) [49]. Growing evidence suggests EBRT may also have a role for bridging or downstaging HCC to facilitate access to resection or transplantation [44,50,51,52,53,54].

Encouraging evidence supports combining systemic therapy with radiotherapy in treating HCC. An international randomized phase III trial (NRG/RTOG1112) compared 177 patients with locally advanced HCC (74% with MVI) treated with SBRT and then sorafenib versus sorafenib alone [55,56]. The median OS of the SBRT and then sorafenib group was 15.8 months compared to 12.3 months for the sorafenib alone arm. Univariate analysis yielded a hazard ratio (HR) of 0.77 (*p* = 0.055), whereas the difference was statistically significant on multivariate analysis (HR 0.72, *p* = 0.04) when adjusted for pre-determined stratification factors. The median PFS with and without SBRT in the experimental arms was 9.2 vs. 5.5 months (*p* < 0.001). Notably, patients with MVI had maintained or reduced tumor burden with SBRT. Furthermore, the addition of SBRT to sorafenib systemic therapy was found to have a comparable safety profile to sorafenib alone with 47% compared to 42% of patients experiencing grade ≥ 3 adverse events, respectively. The difference in OS following univariate analysis was not statistically significant (*p* = 0.055), which was potentially due to early accrual termination and under-powering. Accrual was impacted by ICIs becoming the standard systemic therapy, replacing sorafenib, during trial recruitment. Nonetheless, the probability of SBRT improving OS was determined to be 97%, and the multivariate analysis using randomization stratification factors improved statistical power, thereby supporting the interpretation that SBRT may add a clinically meaningful benefit [57].

Taken together, SBRT has demonstrated comparable or improved local control, OS, and safety profile compared to other locoregional therapies, such as RFA and TACE, in the treatment of various stages of HCC. The results of the NRG/RTOG1112 trial highlight the benefit of combining SBRT and systemic therapy for unresectable HCC, particularly in patients with MVI. Guidelines for the treatment of HCC have shifted toward incorporating EBRT in the treatment algorithms [8,9,58,59,60,61].

## 3. Immunotherapy for Hepatocellular Carcinoma

With the introduction of ICIs, immunotherapy has become the standard of care systemic treatment for advanced HCC. Current ICI therapeutics are monoclonal antibodies that bind and impede immune checkpoint proteins on T cells or cancer cells (Table 1). Examples include programmed death protein 1 (PD-1), programmed death ligand 1 (PD-L1), and cytotoxic T-lymphocyte associated protein 4 (CTLA-4) (Table 1). The therapeutic blockade of PD-L1/PD-1 or CTLA-4/cluster of differentiation 80 (CD80) interactions promotes the re-invigoration of anti-tumor immunity. The ICI therapeutics can be combined with anti-angiogenesis agents, such as bevacizumab (anti-VEGF-A) (Table 2).

Several multicenter phase III randomized control trials have solidified immunotherapy as the first-line standard of care systemic treatment for advanced HCC. In the IMbrave150 trial [62,63,64], the combination of atezolizumab (PD-L1 inhibitor) with bevacizumab (VEGF-A inhibitor) (atezo-bev) was beneficial compared to sorafenib in patients with unresectable HCC. The median OS was 19.2 months for the immunotherapy group and 13.4 months for the sorafenib treatment group (*p* < 0.001) [62,63,64]. In the HIMALAYA trial [65,66], the combination of durvalumab (PD-L1 inhibitor) and tremelimumab (CTLA-4 inhibitor) has demonstrated an improved median OS (16.4 months) over sorafenib (OS 13.8 months) (*p* = 0.004) [65,66]. The Checkmate 9DW trial revealed that nivolumab (PD-1 inhibitor) and ipilimumab (CTLA-4 inhibitor) treatment achieved a median OS of 23.7 months compared to a median OS of 20.6 months with TKI (sorafenib or lenvatinib) treatment (*p* = 0.018) [67]. These immunotherapy regimens had similar grade 3 or 4 adverse event rates compared to the TKI control arms.

Notably, these phase III immunotherapy trials either exhibited modest benefit in patients with MVI or excluded patients with severe MVI into the main trunk of the portal vein. For instance, this high-risk cohort with portal vein MVI derived a slight benefit from atezo-bev immunotherapy over sorafenib (median OS 7.6 months vs. 5.5 months, *p* = 0.104) [68]. When comparing the patients with vs. without main portal vein trunk invasion in the IMbrave150 trial, the median OS was comparatively poor at 7.6 months vs. 21.1 months, respectively. The poor prognosis of patients with HCC MVI, in spite of advancements in immunotherapy options, reflects an unaddressed limitation with current therapeutic approaches.

Although ICIs have improved outcomes in advanced HCC, a minority of patients retained durable treatment response (12.5% to 47% at 36 months), and several patients did not respond to immunotherapies alone (25% to 40%) [64,65,67]. In addition, in real-world settings, patient outcomes have been inferior to those in trial findings [69]. This disparity is potentially attributed to patients having worse liver function, advanced disease, or MVI compared to those eligible for trial participation. These challenges in treatment response highlight the unmet need for strategies to optimize and enhance immunotherapy efficacy in treating HCC.

## 4. Combining Radiotherapy and Immunotherapy for Hepatocellular Carcinoma

**Figure 1:**
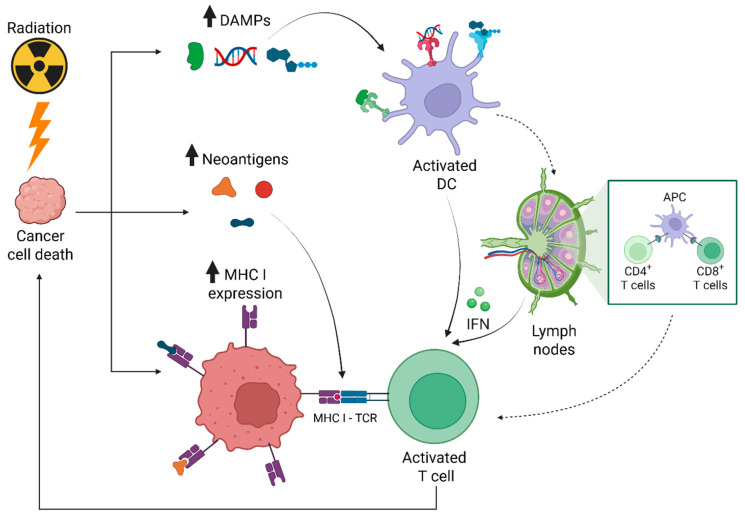
Schematic showing effects of radiation on cancer cells to stimulate an anti-tumor response and create a tumor microenvironment permissive to immunotherapy. Radiation causes DNA damage to cancer cells, stimulates the release of neoantigens and damage-associated molecular patterns (DAMPs), upregulates major histocompatibility complex I (MHCI) expression, and incites immunostimulatory signals. Abbreviations: CD, cluster of differentiation; IFN, interferon; TCR, T cell receptor. Figure adapted from images created with BioRender.com.

The combination of radiation and immunotherapy represents an attractive oncologic treatment approach with the potential for synergistic benefit. Radiation can stimulate an anti-tumor response and potentiate a tumor microenvironment permissive to immunotherapy [70]. Preclinical experiments suggest multifaceted mechanisms (Figure 1), including enhancing the volume and diversity of neoantigen presentation [71,72], releasing damage-associated molecular patterns (DAMPs), upregulating major histocompatibility complex I (MHCI) [73], and sensitizing tumors to immune-mediated killing [74,75,76,77]. Radiation also promotes proinflammatory cytokine signaling [78], inducing immune cell activation, maturation, and recruitment [79,80]. The radiation-associated immune stimulation can be leveraged to enhance immunotherapy effect [81]. Radiotherapy also confers immunosuppression, including upregulating inhibitory immune checkpoints [82], potentially prompting lymphopenia [83], or recruiting immunosuppressive cell populations [84,85,86]. This radiation-related immunosuppression can potentially be downregulated by combining the complementary effects of immunotherapy [78]. With this compelling biological basis and the emergence of immunotherapy in HCC treatment, many recent studies have explored the combination of radiotherapy with immunotherapy (Table 3 and Table 4). Combinations of TARE and ICIs are also under active investigation and have been reviewed separately [87].

Most studies of combining radiotherapy with immunotherapy for HCC have focused on advanced and unresectable HCC. Patients with main trunk portal vein MVI had limited benefit in IMbrave150 and were excluded from the HIMALAYA and Checkmate 9DW trials. Given the demonstrated benefit of EBRT for HCC with MVI, a combination of radiotherapy with immunotherapy could improve the outcomes in patients with advanced MVI.

### 4.1. Radiotherapy and Immunotherapy in Combination Improves Treatment of HCC with MVI (Table 3)

An ongoing phase III randomized trial evaluated patients with HCC MVI into the first branch or main trunk of the portal vein. The patients were treated with either toripalimab (PD-1 inhibitor) plus radiotherapy (*n* = 25) or sorafenib alone (*n* = 11) [88]. The combined immunotherapy and radiation arm demonstrated improved median OS (not reached vs. 10.9 months, *p* < 0.001) and ORR (44% vs. 9%, *p* = 0.041) compared to the sorafenib arm. Similar rates of grade ≥ 3 adverse events were observed: 24% in the immunotherapy and radiotherapy combination arm vs. 36% in the sorafenib arm.

Another ongoing randomized control trial compared patients with portal vein MVI receiving camrelizumab (PD-1 inhibitor) and apatinib (VEGFR2 inhibitor) with (*n* = 40) or without (*n* = 20) SBRT [89]. The addition of SBRT improved median OS (12.7 vs. 8.6 months), median PFS (4.6 vs. 2.5 months), ORR (47.5% vs. 20%), and disease control rate (72.5% vs. 40%) compared to the systemic therapy alone [90]. Grade ≥ 3 treatment-related adverse events were observed in 21.7% of the patients.

Several prospective single-arm phase II trials have also suggested favorable outcomes and safety profiles when combining immunotherapy and radiotherapy to treat patients with HCC MVI (Table 3). Patients with HCC MVI were treated with concurrent EBRT and nivolumab (PD-1 inhibitor) followed by consolidative nivolumab [91]. Median PFS was 5.6 months and median OS was 15.2 months. Grade 3 or 4 adverse events occurred in 12% of the patients. Sintilimab (PD-1 inhibitor) and bevacizumab (VEGF-A inhibitor) combined with EBRT was administered to 46 patients with HCC invading the portal vein [92]. The median OS was 24 months, median PFS was 13.8 months, and ORR was 58.7%. The combination treatment was associated with grade 3 or 4 adverse events in 65% of the participants. Neoadjuvant tislelizumab (PD-1 inhibitor) and EBRT followed by surgery and adjuvant tislelizumab was assessed in HCC patients with MVI [93]. The ORR was 30% and the median OS was 18.7 months, with 13% of patients experiencing grade ≥ 3 treatment-related adverse events. The benefit of combining EBRT and immunotherapy in patients with MVI is also reinforced in retrospective analyses [94,95].

**Table 3:** A summary of studies combining external beam radiotherapy and immunotherapy to treat HCC with MVI.

**Study**	Population and Treatment	Design	*n*	RT Dose	Outcomes			Safety
					mOS	mPFS	ORR	Grade ≥ 3 AEs
Chen et al., IJROBP, 2025 [88]	First branch or main trunk PV MVI; 1st line toripalimab + RT vs. sorafenib	Phase III RCT	25 vs. 11	40–60 Gy in 10 fractions	NR vs. 10.9 mo *		44.0% vs. 9.1% *	24.0% vs. 36.4%
Hu, Clin Cancer Res, 2023 [89]	PV MVI; 1st line SBRT + camrel-apa vs. camrel-apa	Phase II RCT	40 vs. 20	36–40 Gy in 5–6 fractions	12.7 vs. 8.6 mo	4.6 vs. 2.5 mo	47.5% vs. 20%	21.7%
Kim et al., JHEP, 2024 [91]	MVI; 1st or 2nd line RT + nivo	Phase II	50	43–50 Gy in 10 fractions	15.2 mo		36%	(TRAEs) 12%
Zhu et al., Hepatology, 2024 [92]	PV MVI; 1st line RT + sintilimab + bevacizumab	Phase II	46	30–50 Gy in 10 fractions	24.0 mo	13.8 mo	58.7%	65.2%
Pan et al., Nat Comm, 2024 [93]	MVI; 1st line tislelizumab + RT → surgery → tislelizumab	Phase II	15	45 Gy in 15 fractions	18.7 mo		30%	13.3%
Su et al., Oncologist, 2024 [94]	PV MVI or tumors > 50% liver volume; 1st or 2nd line atezo-bev + RT vs. atezo-bev	Retro	14 vs. 34	72.6 GyE in 22 fractions	NR vs. 5.5 mo *	5.2 vs. 2.9 mo	50% vs. 11.8% *	(TRAEs) 14.3 vs. 14.7%
Krishnamurthy et al., Adv Radiat Oncol, 2025 [95]	PV MVI; any line RT + ICI	Retro	62	40 Gy in 5 fractions or 45 Gy in 15 fractions	7.7 mo	3.7 mo		1.6%

* denotes *p* value < 0.05. Abbreviations: adverse event (AE) or treatment-related adverse event (TRAE), atezolizumab and bevacizumab (atezo-bev), camrelizumab and apatinib (camrel-apa), gray (Gy), immune checkpoint inhibitor (ICI), median overall survival (mOS), median progression-free survival (mPFS), not reached (NR), nivolumab (nivo), objective response rate (ORR), macrovascular invasion (MVI), portal vein (PV), randomized control trial (RCT), retrospective analysis (Retro), sample size (*n*), stereotactic body radiotherapy (SBRT), radiotherapy (RT).

### 4.2. Radiotherapy and Immunotherapy in Combination Improves Treatment of Unresectable or Advanced HCC (Table 4)

Many studies have evaluated radiotherapy and immunotherapy in combination for the treatment of unresectable or advanced HCC, which was not limited to patients with MVI (Table 4). A single-arm phase II trial treated 21 patients with unresectable HCC using SBRT and camrelizumab (anti-PD-1) [96]. The median PFS and OS were 5.8 and 14.2 months, respectively. Grade 3 treatment-related adverse events were observed in 24% of patients, and there were no grade 4 or 5 adverse events. In the PEMRAD trial, 18 patients with advanced HCC, who progressed on sorafenib, were treated with pembrolizumab and SBRT [97]. The ORR was 41%, the median PFS was 5.4 months, and the median OS was 12.6 months. Grade ≥ 3 treatment-related adverse events occurred in 28% of the patients. A multicenter phase I trial investigated 13 patients with unresectable HCC. They were treated with SBRT and subsequent immunotherapy—either ipilimumab–nivolumab or nivolumab alone [98]. The ipilimumab–nivolumab with SBRT arm had favorable outcomes with improved median PFS (11.6 vs. 2.7 months, *p* < 0.05) and median OS (41.6 vs. 4.7 months, *p* < 0.05). Grade 3 adverse events occurred in 50% of patients in the nivolumab arm and 71.4% of the patients in the ipilimumab–nivolumab arm. Grade 3 hepatotoxicity occurred more often with the ipilimumab–nivolumab combination (42.9%) than the nivolumab arm (16.7%). No grade 4 or 5 adverse events were observed.

In a single-arm phase II trial, the sequential administration of TACE, SBRT, and then avelumab converted 18 of 33 patients to suitable for curative resection or RFA. Grade ≥ 3 adverse events were experienced in 11 of the 33 patients [99]. Of a larger, 63-patient cohort, 29 (46%) achieved complete response with a 3-year local control rate of 90.5% [100]. Grade ≥ 3 adverse events were observed in 23.8% of the patients.

Retrospective studies have also demonstrated therapeutic benefit in patients with unresectable or advanced HCC receiving radiotherapy and immunotherapy (Table 4). These studies included a combination of radiation with ICIs [101,102]; radiation with ICIs and angiogenesis inhibitors [103,104,105,106,107]; and radiation with ICIs and TKIs [108,109,110,111].

Patients with advanced HCC were treated with ICIs and anti-angiogenic therapy with or without radiotherapy [105]. The addition of EBRT improved ORR (75.9% vs. 24.1%, *p* < 0.001), disease control rate (100% vs. 75.9%, *p* = 0.005), median PFS (8.3 months vs. 4.2 months, *p* < 0.001), and median OS (not reached at 15.5 months vs. 9.7 months, *p* = 0.002). No significant difference in grade 3 or 4 treatment-related adverse events was observed between the two groups. A retrospective analysis of 108 patients with stage IV or recurrent HCC that received radiotherapy and immune checkpoint blockade demonstrated an ORR of 75%, median OS of 17 months, and median PFS of 12.6 months. Grade ≥ 3 adverse events were noted in 30.6% of the cohort [109]. An observational study assessed 36 patients with advanced HCC; they already received ICIs and then received EBRT to treat enlarging or new lesions [110]. The median PFS was 7.4 months, the median OS was 18.8 months, the ORR was 38.9%, and grade 3–4 adverse events occurred in 38.9% of the patients.

**Table 4:** A summary of studies combining external beam radiotherapy and immunotherapy to treat unresectable or advanced HCC.

Study	Population and Treatment	Design	*n*	RT Dose	Outcomes			Safety
					mOS	mPFS	ORR	Grade ≥ 3 AEs
Li et al., Hepatol Int, 2022 [96]	Unresectable; ≥2nd line SBRT + camrelizumab	Phase II	21	30–50 Gy in 10 fractions	14.2 mo	5.8 mo	52.4%	(TRAEs) 23.8%
O’Kane et al., JHEP 2026 [97]	After progression on sorafenib; SBRT + pembrolizumab	Phase II	18	25–50 Gy in 5 fractions	12.6 mo	5.4 mo	41%	(TRAEs) 28%
Juloori et al., IJROBP, 2023 [98]	Unresectable; 1st or 2nd line SBRT + nivo + ipi vs. SBRT + nivo	Phase I	7 vs. 6	40 Gy in 5 fractions	41.6 vs. 4.7 mo *	11.6 vs. 2.7 mo *	57% vs. 0% *	71.4% vs. 50%
Chiang et al., Lancet Gastro Hepatol, 2023 [99]	Locally advanced; 1st line TACE + SBRT + avelumab	Phase II	33	27.5–40 Gy in 5 fractions			42% rCR	33%
Chiang et al., JAMA Oncol, 2024 [100]	Advanced; 1st line TACE + SBRT + avelumab achieved rCR	Secondary analysis	29	27.5–45 Gy in 5 fractions	3 year OS: 75.5%			23.8%
Chiang et al., Liver Cancer, 2023 [101]	Unresectable; 1st line SBRT + ICI vs. SBRT	Retro with PSM	30 vs. 70	25–45 Gy in 5 fractions	1 year OS: 92% vs. 74%		88% vs. 50% *	(TRAEs) 35% vs. 22.5%
Chiang et al., Front Oncol, 2021 [102]	Unresectable; 1st line SBRT + ICI vs. TACE	Retro with PSM	16 vs. 48	25–37.5 Gy in 5 fractions	1 year OS: 93.8% vs. 80.4% *	1 year PFS: 93.3% vs. 77.8% *	87.5% vs. 16.7% *	18.8% vs. 60.4% *
Hsu et al., Chin Med J, 2024 [103]	Advanced; any line RT + ICI vs. ICI	Retro with PSM	71 vs. 71	20–60 Gy at 1.8–10 Gy per fraction	20.9 vs. 11.2 mo *	5.7 vs. 2.9 mo *	40.8% vs. 19.7% *	9.5% vs. 9.1%
Su et al., Front Immunol, 2022 [104]	Advanced; any line RT + ICI + AA vs. ICI + AA	Retro with PSM	40 vs. 40	48 Gy in 16 fractions	18.5 vs. 12.6 mo *	8.7 vs. 5.4 mo *	40% vs. 25%	Not stat different
Ning et al., IJROBP, 2024 [105]	BCLC C; 1st line ICI + AA + RT vs. ICI + AA	Retro with PSM	29 vs. 29	30–60 Gy in 8–30 fractions	NR vs. 9.7 mo *	8.3 vs. 4.2 mo *	75.9% vs. 24.1% *	36.2% in entire cohort
Nakabori et al., Curr Oncol, 2024 [106]	Advanced; Atezo-bev + locoregional therapy vs. atezo-bev	Retro	10 vs. 19	40–50 Gy in 10 fractions	NR vs. 19.8 mo *	NR vs. 7.4 mo *	80.0% vs. 21.1% *	40% vs. 37%
Manzar et al., Cancers, 2022 [107]	Advanced; any line Atezo-bev + RT	Retro	21	20–75 Gy in 5–25 fractions	16.1 mo			
Wang et al., IJROBP, 2024 [108]	Unresectable; 1st line SBRT + Lenvatinib + ICI vs. SBRT + lenvatinib	Retro	146 vs. 68	40–52.5 Gy in 5–10 fractions	31.2 vs. 17.4 mo *	15.6 vs. 8.8 mo *	63% vs. 39.7% *	Not stat different
Zhai et al., Front Immunol, 2025 [109]	Stage IV or recurrent; any line RT + ICI	Retro	108	50–65 Gy in 20–30 fractions	17.0 mo	12.6 mo	75.0%	30.6%
Ning et al., Hepatobil Surg Nutr, 2023 [110]	Advanced; RT after progression on ICI	Retro	36	30–70 Gy in 5–30 fractions	18.8 mo	7.4 mo	38.9%	38.9%
Zhong et al., Front Oncol, 2021 [111]	BCLC C; any line RT + ICI + AA	Retro	16	30–60 Gy at 1.8–4 Gy per fraction	20.9 mo	4.6 mo	40%	25%

* denotes *p* value < 0.05. Abbreviations: adverse event (AE) or treatment-related adverse event (TRAE), anti-angiogenesis agent (AA), atezolizumab and bevacizumab (atezo-bev), gray (Gy), immune checkpoint inhibitor (ICI), median overall survival (mOS), median progression-free survival (mPFS), not reached (NR), nivolumab (nivo), ipilimumab (ipi), objective response rate (ORR), propensity score matching (PSM), retrospective analysis (Retro), sample size (*n*), stereotactic body radiotherapy (SBRT), radiotherapy (RT), radiological complete response (rCR), Barcelona Clinic Liver Cancer stage C (BCLC C).

In a propensity score-matched investigation, patients with intermediate or advanced HCC receiving a PD-1 inhibitor, anti-angiogenic agent, and EBRT were compared to a control group receiving a PD-1 inhibitor and anti-angiogenic agent [104]. The study demonstrated statistically significant improvement in median OS (18.5 vs. 12.6 months, *p* = 0.043) and median PFS (8.7 vs. 5.4 months, *p* = 0.013) with the triple therapy vs. systemic therapy alone. There was no significant difference in adverse events between the treatment arms. Another study retrospectively compared patients with advanced HCC treated with EBRT and anti-PD-1 therapy or anti-PD-1 monotherapy [103]. Investigators found improved median PFS (5.7 vs. 2.9 months, *p* < 0.001), median OS (20.9 vs. 11.2 months, *p* = 0.018), and ORR (40.8% vs. 19.7%, *p* = 0.006) with the addition of radiotherapy without increasing toxicity. Patients with unresectable locally advanced HCC who received either SBRT combined with immunotherapy (16 patients) or radiotherapy alone (48 patients) were compared using propensity score-matching analysis [102]. The 12-month PFS (93.3% vs. 16.7%, *p* < 0.001), 24-month PFS (77.8% vs. 2.1%, *p* < 0.001), 12-month OS (93.8% vs. 31.3%, *p* < 0.001), 24-month OS (80.4% vs. 8.3%, *p* < 0.001), and grade ≥ 3 adverse events (18.8% vs. 60.4%, *p* = 0.004) all favored the SBRT with immunotherapy group.

In retrospective analyses, the addition of locoregional treatments, such as SBRT, to atezo-bev improved outcomes [106]. When compared to 19 patients receiving atezo-bev alone, the median PFS (not reached vs. 7.4 months, *p* = 0.004) and median OS (not reached vs. 19.8 months, *p* < 0.001) were improved in the group receiving liver-directed treatment along with atezo-bev. A retrospective study of patients with unresectable HCC treated with combination therapies evaluated 146 patients that received SBRT, lenvatinib, and a PD-1 inhibitor as well as 68 patients that received SBRT and lenvatinib [108]. Outcomes were significantly improved in the group receiving triple therapy including immune checkpoint inhibition compared to the dual therapy group—for example, median OS (31.2 vs. 17.4 months, *p* < 0.001) and median PFS (15.6 vs. 8.8 months, *p* < 0.001). The incidence of any grade as well as grade ≥ 3 treatment-related adverse events were not statistically different between the triple and dual therapy groups.

## 5. Ongoing Trials Combining Radiotherapy and Immunotherapy for Hepatocellular Carcinoma (Table 5)

Recognizing the unmet need for evaluating the combination of EBRT and immunotherapy to treat HCC, several clinical trials are underway. Table 5 summarizes examples of 24 relevant studies listed in clinicaltrials.gov as of February 2026. For example, a multicenter phase III randomized trial, coordinated by NRG (NRG-GI012, HELIO-RT, NCT07166406), compares immunotherapy with or without combination with radiotherapy. The treatment arm 1 includes atezo-bev, durvalumab-tremelimumab, or ipilimumab-nivolumab, whereas treatment arm 2 combines SBRT with these immunotherapies (Table 5). This study aims to elucidate the benefit of combining radiotherapy with immunotherapy.

Other ongoing trials cover a wide range of radiotherapy and immunotherapy combinations. The therapeutic agents for systemic therapy include combinations of (i) ICIs, such as anti-PD-1, anti-PD-L1, and anti-CTLA-4 antibodies; (ii) angiogenesis inhibitors, such as VEGF-A and VEGFR inhibitors; and (iii) TKIs. The primary outcomes include OS, PFS, ORR, and downstaging efficacy (Table 5).

**Table 5:** Ongoing clinical trials combining external beam radiotherapy and immunotherapy to treat HCC.

Study, Clinicaltrials.gov ID	Patient Population	Design	Estimated Enrollment	Treatment	Primary Outcome
NRG-GI012, HELIO-RT, NCT07166406	Macrovascular invasion	Phase III, Randomized control trial	252	Arm 1: ICI (atezo-bev, durva-treme, or ipi-nivo) Arm 2: SBRT with ICI (atezo-bev, durva-treme, or ipi-nivo)	OS
NCT07293468	Unresectable	Phase II/III	106	TACE then SBRT followed by atezo-bev vs. Y90 radioembolization followed by atezo-bev	PFS
HSBRT2402, NCT06313190	Unresectable	Phase II, Randomized control trial	140	SBRT vs. SBRT with adjuvant sintilimab (anti-PD-1)	PFS
NCT06828380	Advanced with vascular invasion	Phase II, Randomized control trial	120	ICI vs. ICI and proton radiation	PFS
NCT06592612	Oligo-progression after first-line systemic therapy	Phase II, Randomized control trial	70	Second-line systemic therapy vs. SBRT with second-line systemic therapy	PFS
NCT07179900	Unresectable	Phase II, Randomized control trial	60	Sintilimab with bev vs. EBRT with bev and QL1706 (anti-PD-1 and anti-CTLA-4 bispecific antibody)	ORR and R0 resection rate
HSBRT2401, NCT06261125	Abdominal metastatic lymph nodes without other sites of extrahepatic metastases	Phase II	60	SBRT followed by adebrelimab (anti-PD-L1) and lenvatinib. Arm A: patients that did not previously receive PD-1/PD-L1 antibodies. Arm B: patients that progressed on PD-1/PD-L1 antibodies.	PFS
NCT07062055	BCLC C with portal vein tumor thrombus or oligo-metastasis	Phase II, Single arm	46	SBRT with bev and QL1706	PFS
NCT06999707	BCLC B or C	Phase II, Single arm	45	EBRT (conventional or stereotactic doses) with durva-treme	PFS
NCT06605664	Unresectable, BCLC B or C	Phase II, Diagnostic	45	Hyperpolarized 13C pyruvate MRI before and 2–5 weeks after radiotherapy combined with atezo-bev	Dynamic Nuclear Polarization
NCT05917431	Unresectable or oligo-metastatic	Phase II, Single arm	39	SBRT with tislelizumab (anti-PD-1) and regorafenib (TKI)	PFS
NCT06524466	Resectable with macrovascular invasion	Phase II, Single arm	35	Neoadjuvant SBRT with lenvatinib and pucotenlimab (anti-PD-1) then surgical resection	ORR and treatment complete rate
NCT04988945	Unresectable	Phase II, Single arm	33	TACE and SBRT followed by durva-treme	Downstaging for resection
NCT06434480	Oligo-progression on atezo-bev	Phase II, Single arm	30	SBRT then continuing atezo-bev	PFS
IMMULAB, NCT03753659	Advanced	Phase II	30	Peri-ablation pembrolizumab (anti-PD-1) with either RFA, MWA, or brachytherapy, with or without TACE	ORR
NCT04430452	Advanced	Phase II	21	Arm I: hypofractionated RT with durva Arm II: after progression on prior PD-L1 therapy, hypofractionated RT with durva-treme Arm III: No prior PD-L1 therapy, hypofractionated RT with durva-treme	ORR
NCT03942328	Unresectable HCC or intrahepatic cholangio-carcinoma	Phase I/II, Single arm	85	SBRT followed by autologous dendritic cells, pneumococcal vaccine, and atezo-bev	Toxicity and PFS
NCT06725121	Advanced with macrovascular invasion	Phase I/II, Single arm	48	SBRT followed by atezo-bev	Downstaging efficacy for liver transplant
NCT05286320	Advanced with portal vein tumor thrombus	Phase I/II, Single arm	27	SBRT followed by pembrolizumab and lenvatinib	Safety rate and ORR
NCT07305428	Unresectable	Pilot, Single arm	30	SBRT followed by ICI	Rate of successive tumor downstaging
NCT07091942	Unresectable that achieve partial response to atezo-bev	Observational	1600	Cohort 1: loco-regional treatment (including definitive RT, surgical resection, or RFA) Cohort 2: no further treatment	Recurrence-free survival
NCT06408753	Unresectable	Observational	50	ICI vs. ICI with SBRT	Exosomal serum PD-L1 level and immune profile of peripheral blood mononuclear cells
NCT07230080	Unresectable	Observational	17	TACE followed by SBRT and ICI	ORR
NCT06639971	Advanced, unresectable	Retrospective cohort	500	RT with ICI and targeted therapy vs. ICI and targeted therapy alone	PFS

Abbreviations: atezolizumab and bevacizumab (atezo-bev), Barcelona Clinic Liver Cancer (BCLC), bevacizumab (bev), durvalumab (durva), durvalumab and tremelimumab (durva-treme), immune checkpoint inhibitor (ICI), ipilimumab and nivolumab (ipi-nivo), microwave ablation (MWA), progression-free survival (PFS), objective response rate (ORR), RFA (radiofrequency ablation), SBRT (stereotactic body radiotherapy), TACE (transarterial chemoembolization).

## 6. Concluding Remarks and Future Perspectives

There is growing interest in exploring the benefit of combining radiotherapy with immunotherapy in advanced or unresectable HCC for prolonging survival and the possibility of downstaging. Considerations for studies investigating the use of radiotherapy with immunotherapy in treating HCC include ensuring a safe combination of the treatment modalities. There is evidence that anti-angiogenic agents can exacerbate SBRT related toxicity with reports of up to 3% risk of bowel perforation 4 months after completion of SBRT [112,113,114]. A small single-center study showed no severe gastrointestinal toxicities when combining bevacizumab with SBRT to colorectal liver metastases [115]. A joint consensus statement with the European Society for Medical Oncology (ESMO) and the European Society of Radiotherapy and Oncology (ESTRO) was recently published that details recommendations when combining radiotherapy with ICIs, VEGF inhibitors, or TKIs [116]. The consensus statement describes the addition of radiotherapy to PD-1 or PD-L1 ICIs as generally safe and recommends discretion when using CTLA-4 ICIs with head and neck or abdominopelvic SBRT. An exercise of caution is recommended when combining EBRT with anti-VEGF antibodies or TKIs. Furthermore, the concurrent use of anti-VEGF antibodies or TKIs with thoracic SBRT and TKIs with liver SBRT was not recommended. Albeit, seminal work examining the combination of systemic therapy with liver SBRT, including the NRG/RTOG1112 trial [117], were not referenced in this joint consensus statement.

Immunotherapies and radiotherapy can precipitate distinct etiologies of hepatotoxicity. ICIs, particularly PD-1/PD-L1 and CTLA-4 combinations, can trigger an aberrant immune targeting of normal liver tissue [67,98]. This immune-mediated adverse event generally presents as asymptomatic transaminitis. Management can involve ICI cessation, steroids, and occasionally immunosuppressants. With modern techniques, radiation-induced liver disease (RILD) is a rare phenomenon. Classic RILD involves anicteric abdominal pain, hepatomegaly, and elevated alkaline phosphatase, whereas non-classic RILD presents with transaminitis. The treatment of RILD involves supportive measures. Taken together, there may be a heightened risk of adverse effects with combining systemic therapies and SBRT, warranting deliberate trial design and careful consideration for toxicity mitigation.

The optimal timing of immunotherapy–radiotherapy combinations remains to be elucidated. To date, no clinical trials have directly compared the concurrent versus sequential administration of these treatment modalities. Preclinical studies in murine models have demonstrated benefit in the various sequencing of radiation and immunotherapy [118,119,120]. With the exception of Keynote A-18 [121], several phase III studies exploring conventionally fractionated EBRT for the definitive treatment of lung [122,123], head and neck [124,125,126,127], and gynecologic cancers [128] have demonstrated no benefit with concurrent ICIs. Benefit has been demonstrated in phase III trials with the sequential administration of conventionally fractionated EBRT followed by ICIs [129,130,131]. In the setting of SBRT ablative doses and ICI combinations, phase III trials are ongoing, and phase I and II studies have shown promising benefit with concurrent or sequential treatments [132,133,134]. Among the investigations of radiotherapy–immunotherapy combinations to treat HCC herein detailed, concurrent [91,92,93,96,97] as well as sequential (SBRT followed by ICI) [98,99,100] approaches have demonstrated favorable outcomes. The remaining studies discussed do not specify and may represent a heterogeneous sequencing of the radiotherapy and immunotherapy for HCC treatment. Although NRG/RTOG 1112 did not examine immunotherapy, this trial demonstrates the benefit and safety of delivering SBRT and then systemic therapy. Overall, the ideal sequencing of EBRT and ICI treatment modalities to treat HCC has yet to be established.

Investigations into the combination of immunotherapy and radiotherapy could benefit from biomarker analysis to elucidate putative population heterogeneity. In principle, meaningful biomarkers will enable the selection of appropriate patients more likely to benefit from ICI treatment [135,136]. Post hoc analysis of the large phase III trials evaluating atezo-bev and nivolumab in advanced HCC demonstrated putative predictive biomarkers for selecting patients more likely to respond to immune checkpoint inhibition [137,138]. Potential predictive biomarkers include a low ratio of neutrophils to lymphocytes, low ratio of platelets to lymphocytes, low AFP, elevated effector T-cell populations in tumor tissue, and enriched T-cell effector inflammation pathways and gene signatures. To date, there are no validated or standardized biomarkers to predict HCC treatment response to immune checkpoint blockade.

In the emerging landscape of HCC treatment approaches, the further exploration of radiotherapy and immunotherapy is warranted and necessitates study design that is mindful of preserving safety as well as interrogating predictive and prognostic factors from the population to the molecular level. Growing development in HCC treatment options warrants future investigations into the combination of external beam radiotherapy and immune checkpoint inhibition with the judicious incorporation of biomarker analyses.

## Figures and Tables

**Table 1:** Immune checkpoint proteins and immune checkpoint inhibitors (ICIs) used to treat HCC.

Immune Checkpoint Proteins	Immune Checkpoint Inhibitors (ICIs)
PD-L1	Atezolizumab (atezo), Durvalumab (durva), Avelumab
PD-1	Nivolumab (nivo), Pembrolizumab (pembro), Toripalimab, Sintilimab, Camrelizumab, Tislelizumab
CTLA-4	Ipilimumab (ipi), Tremelimumab (treme)

Bolded text indicates drug target protein and drug class.

**Table 2:** Angiogenesis inhibitors and tyrosine kinase inhibitors (TKIs) used to treat HCC.

Angiogenesis Proteins	Angiogenesis Inhibitors
VEGF-A	Bevacizumab (bev)
VEGFR2	Apatinib
**Tyrosine Kinases**	**Tyrosine Kinase Inhibitors (TKIs)**
Tyrosine kinases	Sorafenib, Lenvatinib, Cabozantinib

Bolded text indicates drug target protein and drug class.

## Data Availability

No new data were created in this study. Data sharing is not applicable to this paper.
